# Rethinking Intrusiveness: Exploring the Sequential Organization in Interactions Between Infants and Mothers

**DOI:** 10.3389/fpsyg.2019.01543

**Published:** 2019-07-24

**Authors:** Valentina Fantasia, Laura Galbusera, Corinna Reck, Alessandra Fasulo

**Affiliations:** ^1^Department of Social and Developmental Psychology LInC-Interaction & Culture Laboratory, Sapienza University of Rome, Rome, Italy; ^2^Max-Planck-Institut für Bildungsforschung, Berlin, Germany; ^3^Inter-self Lab, Institut für Philosophie, Literatur, Wissenschaft und Technologiegeschichte, Geistes- und Bildungswissenschaften, Technische Universität Berlin, Berlin, Germany; ^4^Hochschulklinik für Psychiatrie und Psychotherapie der Medizinischen Hochschule Brandenburg, Immanuel Klinik Rüdersdorf, Rüdersdorf, Germany; ^5^Department of Psychology, Ludwig-Maximilians University, Munich, Germany; ^6^Department of Psychology, University of Portsmouth, Portsmouth, United Kingdom

**Keywords:** intrusiveness, interactional rhythms, postpartum depression, preliminaries, sequential organization

## Abstract

To date, studies investigating maternal postpartum depression (PPD) have mainly focused on identifying failures in interactions of postpartum depressed mothers and their infants, often attributed to single dysfunctional maternal behaviors. Intrusiveness has been identified as a dysfunctional behavior characterizing mothers suffering from PPD. However, this research does not consider the co-constructed and sequential nature of social interactions, in which single behaviors cannot be conceived as isolated or disconnected units. The aim of the work presented in this paper was to explore the interactional dynamics underlying maternal behaviors previously identified as intrusive by mainstream literature on postpartum depression. Through a conversation analytical approach, we analyzed filmed interactions between mothers with and without postpartum depression and their 3-months-old infants. The analyses of 4 selected episodes illustrate similar dyadic activities, yet presenting different levels of mutuality and affective attunement. Results showed two normative features of social interactions that contributed to the different quality in the mutual adjustment of the partners: interactional rhythm and preliminaries. Interactional rhythm refers to the structuring of infants' spontaneous activity into a turn sequence, whereas preliminaries consist of verbal or nonverbal moves that anticipate following action. As evident from our analytical observations, what seems to be hindering the mutual coordination (previously labeled as “intrusive”) is not based on specific individual behaviors but on the absence or violation of such interactional norms. Adopting an interactive and dynamical framework, we shifted the focus from maternal behaviors considered as dysfunctional to observing the unfolding of interactional aspects contributing to better or poorer sequential structuring. We argue that these aspects shape the possibilities for the infant's participation. Finally, we discuss the theoretical and methodological implications of adopting a conversation analytical approach for a better understanding of the relational dynamics related to clinical and non-clinical interactions.

## Introduction

Since early after birth, infants are immersed in a world of social exchanges and affective interactions with their caregivers. Affective synchrony (Gratier and Devouche, 2011) and interactional coordination are two dimensions that have been recognized as essential for the infant to engage in early, mutually regulated interactions with adults, and more generally for communicative and social development (Jaffe et al., 2001; Trevarthen and Aitken, 2001; Lamb et al., 2002; Stern, 2002; Gratier, 2003; Tronick and Beeghly, 2011). Mutual regulation, however, is not an all-or-nothing property of interactions but rather a dynamic, moment my moment achievement reflecting the quality of mutual alignment and dis-alignments between participants as the interaction unfolds. Mutual regulation is therefore influenced by many different factors and circumstances, e.g. the infant's affective, social and cognitive growth. On the caregiver's side, for instance, clinical conditions may be hindering the possibility to experience mutual and affectively charged intersubjective exchanges, as in the case of postpartum depression (PPD). A long tradition of research has identified PPD as a clinical condition that may affect the quality of mother-infant early interactions, generally involving maternal affective withdrawal or intrusiveness as primary behavioral dimensions (Reck et al., 2017).

The aim of the work presented in this paper is to explore the interactional dynamics underlying maternal behaviors previously identified as intrusive. In the following sections of the paper we first introduce the concept of dyadic mutual regulation within the field of early infant-caregiver interactions, to then move on to discuss the circumstances in which this regulation process might be affected or altered, as in the case of postpartum depression. A theoretical revision of intrusiveness as an interactional phenomenon is then advanced, proposing an analytical approach that focuses on the structural aspects supporting (very early) interactions. By using a conversation analytical approach, filmed interactions between infants and mothers (with and without depression) participating in a study adopting a still-face paradigm were analyzed, enabling the systematic observation of two aspects as illustrative of the way even very early interactions are sequentially structured and ordered: *interactional rhythms* and *preliminaries*. We discuss these findings suggesting that traditional measures of intrusiveness fail to take into account the sequential relevance and organization of maternal actions, in relation to the actions of the infant. On the contrary, the sequential analysis applied can help clarify the interactional structures and dynamics underlying what has been so far identified as “intrusive maternal behavior” and thus set the ground for rethinking the very notion of intrusiveness within a more relational framework.

### Sequential Organization and Mutual Regulation: Key Aspects for Studying Infant-Caregiver Interactions

Over the past fifty years evidence from research on social, developmental and educational psychology have demonstrated the dynamical nature of early non-verbal interactions by focusing on the way caregivers (mostly mothers) and infants are mutually responsive to each other's movements, speech and affective displays. This evidence has supported a new conceptualization of caregivers-infants interactions as cooperative and jointly constructed (Trevarthen and Hubley, 1978; Trevarthen, 1998), contrasting previous theoretical understanding of infants, especially in the first months, as passive and unintentional interactants. The co-constructed nature of early interactions was also emphasized by the Mutual Regulation Model (MRM, Tronick and Cohn, 1989; Tronick and Weinberg, 1997), one of the most well-established theoretical accounts of early intersubjective interactions. This model describes mother-infant interactions as patterns of moment-to-moment mutual adjustments that move from states of affective coordination and matching to states of affective dis-coordination and disengagement. Coordination and synchrony between the infant and the caregiver are not steady, but rather a complex, dynamic flow, where dis-coordinated moments are considered as normal interactive dis-alignment, usually followed by successful affective reparations (Tronick and Weinberg, 1997). Interaction is thus described as a structured system of mutually regulated units of behavior, as each partner's behavior is influenced and coordinated through the behavior of the other (Tronick et al., 1979; Cohn and Tronick, 1988). Based on this theory, Tronick et al. (1980) developed a scoring system called Monadic Phase System (MP) which captures behavioral dimensions of the mother and the infant such as gaze direction, vocalizations, facial expressions, head orientation and body position, and combines them into macro-categories called *monadic phases*. This instrument has been widely used in infant research.

More recently, observation-oriented infant studies have started looking at mother-infant communication through the lens of structural and conventional elements regulating adult communication. Thanks to the intrinsically dialogic nature of the methodology adopted, infants' behaviors (laughing, crying, gazing) have been identified not only (and always) as responses to the adult's move, but also as interactional initiatives (Trevarthen, 1977; Trevarthen and Hubley, 1978; Reddy and Uithol, 2015) upon which caregivers contingently act, treating them as turns in conversation-like sequences (Berducci, 2010; Rohlfing and Nomikou, 2014). Developmental research has long recognized the importance of early caregiver-infant exchanges structured as repetitive coordinated activity, so called “interaction formats” (Bruner, 1985) or social routines. Changing diaper (Nomikou and Rohlfing, 2011), performing a nursery-rhyme song (Fantasia et al., 2014), playing peak-a-boo (Nomikou et al., 2017), reading a book (Rossmanith et al., 2014) are social routines where the infant's participation is shaped by means of and through such highly familiar sequences. These routines present regularities essential in orienting the infants' behaviors toward established interactional practices and conventions (Leonardi et al., 2016) shaping the infant's emerging participation (Berducci, 2010; Fantasia et al., 2014, 2016). They therefore constitute contexts “in which to observe the process of shaping agentivity, because infants are treated as participants from early on” (Nomikou et al., 2017, p. 2). Participating in daily practices with more experienced speakers is also an essential moment of being socialized to the different aspects regulating more mature, sequentially-organized interactions, such as sharing the attentional focus, orienting toward the speaker or listener, taking turn after a silence, repairing misunderstandings. Within the field of conversation analytical methods, recent studies, very limited still, have revealed that early communicative exchanges are partially supported by some of the principles of interactional order active throughout adult life, such as turn-taking (Berducci, 2010), maximum standard silences (Hilbrink et al., 2015) and overlapping phenomena (Domingueza et al., 2016).

However, there are conditions that impact the continuity, frequency or quality of moments of mutual recognition and contact between infants and caregivers. On the infant's side, autism spectrum disorder has been recognized as a neurodevelopmental condition strongly affecting young children's possibility of intersubjective engagement with their caregivers. On the caregiver's side, postpartum depression (PPD) is one of the conditions best known to negatively influence the quality of interaction of mothers with their infant, including the mutual regulation of affects (Tronick and Weinberg, 1997; Reck et al., 2004). According to the DSM-5 (American Psychiatric Association, 2013), PPD is a psychological disorder that affects around 10% of new mothers (Cooper and Murray, 1997) and has been associated with later difficulties in children's emotional, cognitive and self-regulatory capacities (Field, 1984; Murray, 1992; Murray and Cooper, 1997; Lovejoy et al., 2000; Reck et al., 2017). Two behavioral dimensions have been proposed as critical with regard to the mother's depressive condition: withdrawal and intrusiveness. In the next section we focus on the latter, its theoretical background, methodological applications and limitations.

### Intrusiveness as Disruption of Mutual Regulation

The term intrusiveness is broadly used to describe behaviors causing undesired disruption or annoyance. Intrusive behaviors can involve physical or verbal actions, and can be experienced as affecting less visible dimensions, such as a violation in the sense of the self, and being therefore experienced as unwelcome or uninvited (Oxford Dictionary, 2019). Educational and developmental research has focused extensively on parental behaviors identified as intrusive across different contexts, ranging from studies linking parental beliefs and practices with children's emotional and cognitive development (Mortensen and Barnett, 2019) or schooling outcomes (Grolnick and Pomerantz, 2009; Liew et al., 2018), to the investigation of ethnic, social and economic factors influencing this aspect of parent-child interaction (Ispa et al., 2004). Although a recent study has proposed to look at intrusive behaviors also on the father's side (Olsavsky et al., 2019), intrusiveness seems to be considered primarily as a maternal characteristic, where the mother “has her own agenda in mind as she either overwhelms the child with excessive stimulation or interrupts the child's self-initiated activity to stop it or change its course” (Ispa et al., 2004, p. 1614).

This is particularly evident in infant research, where intrusiveness has been investigated by comparing healthy and clinical mothers. Initially, intrusive maternal behaviors have been described in terms of over-control and under-control (Ricks, 1981), and later reframed as over-stimulation and directiveness (Pine, 1992). Later on, some studies have re-assessed a set of maternal behaviors, initially included in the Monadic Phase Paradigm (MPP) and not related to intrusiveness, as intrusive. Behaviors such as anger/poke, disengage, elicit, play, originally described by the MPP, have been aggregated into macro-categories such as “disengaged,” “positive,” “mixed” and used to classify behavioral patterns of mothers (Cohn et al., 1986, 1990; Field et al., 1990; Campbell et al., 1995). Intrusive behaviors were characterized by low levels of play and high levels of anger (Cohn et al., 1986, 1990), and lower instances of mutual regulation, particularly in dyads in which the mother had postpartum depression (Cohn and Tronick, 1983; Murray et al., 1996; Reck et al., 2004, 2011; Beebe et al., 2008; Hatzinikolaou and Murray, 2010). Other studies have attributed intrusive character to single maternal behaviors occurring in a given time unit, such as rough handling of the infant, poking, pulling, tickling, interfering manipulation and using a loud tone of voice (Cohn et al., 1990; Malphurs et al., 1996; Diego et al., 2002), and/or an angry tone of voice (Tronick and Weinberg, 1997) and intrusive touch (Beebe et al., 2008).

In comparison to healthy controls, mothers with postpartum depression were found more prone to adopt either withdrawing or intrusive behaviors in the interaction with their infants (Reck et al., 2004; Beebe et al., 2008), presenting increased over-stimulation, negative and aggressive actions (e.g., irritation, anger, rough handling) disrupting affective synchrony and interactional coordination (Cohn and Tronick, 1983; Cohn et al., 1986, 1990; Field et al., 1990; Beebe et al., 2008). Such maternal conducts were found to match a corresponding tendency by the infant toward withdrawal, higher stress arousal and negative affect (Cohn et al., 1986, 1990; Field et al., 1988; Diego et al., 2002; Hatzinikolaou and Murray, 2010).

### Critical Issues With Current Definition and Assessment of Intrusiveness

In an attempt to describe the problematic relationship of a young girl with her clinical mother, Daniel Stern (2002) advanced criticisms to using the construct of intrusiveness as clinical index. He argued that intrusiveness is too large as a behavioral unit, too global and vague for clinical or observation purposes, and unpacking “intrusiveness” into smaller behaviors, such as head turns, gaze aversion or speed of physical approach would instead lead to its better clinical understanding.

In most of the studies just presented, clinical and non-clinical, individual behaviors of the mother and the infant were in the first instance assessed and considered separately and independently of each other. To account for the mutual and contingent nature of the interactions observed, ratings of individual behaviors are subsequently matched together by means of time series analyses (e.g., Field et al., 1990; Beebe et al., 2008). Although presenting undiscussed timing accuracy, the costs for this methodological procedure are relatively high in terms of ecological validity and interpretation of the results as the sequential character of any naturalistic interaction is lost. Indeed, considering a single behavior as analytical unit for identifying intrusiveness implies a lack of consideration for the sequential organization of the interaction, making it difficult, if not impossible, to establish whether the action of the infant or the caregiver is an initiative or a response, or how the two participants' acts might be otherwise sequentially linked; hence assuming that all maternal behaviors are initiatives, without the possibility to establish to what extent the infant's behavior contributes to the mother's (intrusive) actions. Yet, precisely in light of the mutual and affectively coordinated nature of very early interactions, mother's behavior is influenced by the infant as much as the other way round.

Additionally, the self-experience of a given manipulation or vocal stimulation by someone else may vary across different persons or change depending on timing, interactional context and in-the-moment affective state of that same person. As strongly suggested by previous research on language development and socialization (Bateson, 1994; Ochs and Schieffelin, 1994; Duranti, 2000), the same maternal behavior may assume different meanings and functions according to the sociocultural norms and nurturing practices and the specific maternal style (Mead and Macgregor, 1951; Schieffelin and Ochs, 1986; Keller et al., 2004). Losing information on the broader sequence in which target behaviors occur impoverish the interpretation of that very behavior and its impact in the dynamical mutual coordination of the dyad. For example, daily caregiver-infant routines are largely based on physical actions on the body of the infant. Beside the necessary daily care activities, entertaining, and playing with infants implies a certain amount of bodily manipulation, sometimes in the form of control or physical guidance by the adult. Consider for instance early social games played by mothers and infants as early as 3 months of age, where multimodality is a necessary part through which bodily experiences and affects sharing occur: during these games the mother is pulling, poking, shaking, or holding the infant; yet, not only infants react with positive engagement to many of those occurrences, but they may react with distress if some component of these rich stimulatory activities is dropped (Fantasia et al., 2014; Nomikou et al., 2017). Assigning a pre-determined affective quality to individual behaviors result in a loss of analytical (and predictive) power on the impact of those specific behaviors or behavioral sequences on the infant.

Recently, improvements toward a more relational view of intrusiveness have been made by the Infant and Caregiver Engagement Phases, first in its original version (ICEP, Weinberg and Tronick, 1999) and then in the revised one (ICEP-R, Reck et al., 2008/2009). This coding instrument considers intrusive actions as those made “regardless of the infant's behavior,” and characterized by a violation of the infant's autonomy. Examples of such behaviors are anticipating the infant's moves without waiting for the infant's response or interrupting the infant's self-initiated activity in order to pursue her own “program” (Weinberg and Tronick, 1999; Reck et al., 2008/2009). In the ICEP-R then, for the first time the caregiver's intrusive behavior is identified by taking into account the position of both interactants around each single act. Despite this important change, the ICEP-R operational definitions of intrusiveness, such as for example “too loud, too expressive or too close to her child” (Reck et al., 2008/2009, p.4), include consideration of the child but without specifying what dimensions of the child behavior are used by the coder to arrive at their definition of what is “too much.”

Altogether, the issues just presented might account for inconsistencies in the way intrusiveness has been defined and studied in research so far, leading to a general interpretative weakness of this construct (Provenzi et al., 2018) and yielding relatively little definitive results concerning its impact on the infant's development and well-being (as especially revealed by cross-cultural studies, see for instance Ispa et al., 2004).

### Rethinking Intrusiveness: Exploring Mother-Infant Interactions Through a CA-Oriented Approach

If intrusiveness was identified at the behavioral level as a failure in coordination and mutual regulation, a similar interactional dis-alignment should be observed when adopting a different methodological framework. In this work we adopt Conversation Analysis (CA) for examining video-recorded episodes of interactions between clinical (diagnosed with post-partum depression, PPD) and non-clinical mothers, and their 3-months-old infants. CA is a method for studying social interactions developed within the ethnomethodological tradition (Garfinkel, 1967). This approach postulates that social actors use “methods” to make their actions reciprocally intelligible, as they are systematically adopted for the production and interpretation of social conduct. Central to CA is the focus on turn-taking (Sacks et al., 1974) and sequential organization, where each communicative turn is shaped by previous one(s) and creates the context for successive moves (Schegloff, 2007). Various levels of sequential organization contribute to the orderly coordination of social encounters and activities (Stivers, 2012). Lay “methods” for the production of talk-in-interaction include features of speech delivery such as volume, intonation and pace, as well as other communication modalities such as gestures, gaze and body movement. In order to take into account such resources, CA has developed a transcription system that captures features of oral speech (Jefferson, 2004) and multimodal behavior without giving a priori importance to one modality over the other (Mondada, 2016). Such level of detail is oriented to identify the way turns are designed to achieve specific actions (Drew, 2013)—for example a greeting or an offer—and to calibrate interactional dimensions such as alignment and affiliation (Stivers, 2012). The aforementioned characteristics of CA make it suitable to the analysis of early interactions, in which different modalities are mobilized simultaneously around the infant.

Through the adoption of this robust and reliable method, our work aimed at investigating whether the construct of intrusiveness could be further analyzed within a more interactional view. This would entail identifying features of multimodal turns and interactional sequences that would pinpoint how the defining characteristic of intrusiveness, i.e., the restriction of the infants' possibilities for action and participation, comes about. More broadly, we are interested in exploring the compatibility of the mutual regulation paradigm with interactional types of analysis such as CA, and attempt to outline avenues for collaboration with clinical research.

## Method

The data selected and analyzed for this study were part of a larger study conducted by Reck et al. (2011). The original study included 28 mothers with current postpartum depression (PPD, according to the ICD-10), 34 healthy mothers recruited from local maternity clinics, and their 3-months-old infants. All clinical mothers and their children were receiving inpatient treatment at the mother–infant unit of the psychiatric Heidelberg University Hospital. Both inpatient and external dyads were video-recorded in the Babylab of the hospital, as participants to a study involving a Still-face paradigm procedure (Tronick et al., 1979; Weinberg et al., 2006). The Still-face is an experimental paradigm consisting of three phases (of 2 min each in this study). In the initial phase the mother is instructed to freely interact with her infant, seated in front of her in a babyseat. After this, the mother is asked to remain still for the entire duration of the Still-face phase, instructed not to move, show any facial expression or respond in any way to the infant, remaining “completely unresponsive, with a flat expressionless face” (Tronick et al., 1978). Finally, in the reunion phase, mother and infant interact freely again.

The video recording of interactions during the Still Face procedure were coded using the Infant and Caregiver Engagement Phases-Revised (ICEP-R, Reck et al., 2008/2009). The study was approved by the independent ethics committee of the University Medical Faculty, Heidelberg. All subjects gave written informed consent in accordance with the Declaration of Helsinki.

### The Present Study

The present study focuses on the free play during the reunion phase following the Still-face. During this phase, the mother's attempts to recover from the experimentally induced distress are more likely to lead to moments of intrusiveness (Weinberg et al., 2006). An initial phase of exploratory observations of filmed interactional episodes in which the mothers' behaviors were coded as intrusive in the original study was carried out. A first sample of episodes was selected during this phase, including both episodes in which mother and infant appeared as positively engaged as well as episodes in which the infant displayed higher level of disengagement, negative facial expressions or distressed vocalization. Since our aim was to shed light on the characteristic features that could discriminate positive and negative interactional outcomes of similar actions equally labeled as intrusive, we first selected two episodes including behaviors coded as intrusive according to the ICEP-R in the original study, but showing visible positive affective engagement (episodes 1 and 3). We then paired each of these episodes with episodes presenting similar activities or behaviors but visible negative affects (episodes 2 and 4).

All episodes were transcribed with ELAN (Sloetjes and Wittenburg, 2008) a software which allows several distinct lines of transcriptions (e.g., gestures, vocalization, gaze) linked together to the same video or audio data. Participants have been given pseudonyms, and the images of both mothers and infants are displayed as anonymous drawings to ensure confidentiality.

Table 1 summarizes information regarding the selected dyads. This phenomenologically-driven approach to data selection allowed us to target our analytical process on the interactional aspects contributing to the positive or negative quality of engagement and alignment of the participants. Three of the four selected episodes were re-transcribed with the CA notation adapted for multimodality (Jefferson, 2004; Mondada, 2016). Of episode 4 we only have the ELAN transcription as we lost access to the videos, which per confidentiality agreement could only be accessed in the Heidelberg lab. The transcription symbols are described in Appendix A. The images come from split screen video grabs showing mother and child from two different cameras. The images' correspondence to the transcript is indicated in the transcript itself. The same symbol in adjacent lines (^*^, +, or ++) indicates simultaneous occurrence of what follows; r- and l-hand mean right and left hand. English translations of the turns in German are added within the transcript.

**Table 1:** Information on the selected dyads and episodes.

**Episode**	**Affective connotation/outcome**	**ICEP original coding**	**Clinical or non-clinical sample**
1	Positive	Intrusive behaviors	Non-clinical
2	Negative	Non-intrusive behaviors	Clinical
3	Positive	Intrusive behaviors	Non-clinical
4	Negative	Intrusive behaviors	Clinical

## Data Analysis

The CA-informed analysis highlighted two distinctive aspects of the sequential organization that differed in the paired episodes: *interactional rhythms* (Gratier, 2003; Gratier et al., 2015) and *pre-sequences* (Schegloff, 1980, 1988).

### Interactional Rhythms

The following episodes involve two mother-infant dyads engaged in interactions primarily structured around the mother's use of hands movements accompanied by vocal comments. Comparing these episodes, differences in the sequential properties and affective quality of the interactions have emerged even though the activities occurring within them may appear similar on the surface.

#### Episode 1

Extract 1a is the introduction to the hands movement game which is the focus of the comparison in this section, and it begins approximately a minute after the end of the Still face phase. Claire, the mother, had reentered the interaction slowly, touching the infant's feet and talking to him, then gently shaking his wrists. After a few seconds the infant had pulled himself up toward her, and she had drawn him closer and kissed his hands. She had then released him down slowly with a long “*Ah”* sounds, then said the first “*Ja super”* (not shown) with a large waving movement of the arms. As the extract begins, she repeats the same phrase (“*Ja super”*) two more times, while introducing a new movement of her hands.

##### Extract 1a

[MUM: CLAIRE; INFANT: TOM, 3 months. M7: 00.05.17–00.05.40]

The transcription symbols in the extracts are described in Appendix A. The images come from split screen video grabs showing mother and child from two different cameras. The images' correspondence to the transcript is indicated in the transcript itself. The same symbol in adjacent lines (^*^, +, or ++) indicates simultaneous occurrence of what follows; r- and l-hand mean right and left hand. English translations of the turns in German are added within the transcript.

**Table:** 

CLA:	*.Hh::a: *Lifts both hands up, palms open (Figure 1a*)*	
2.	TOM:	* Moves arms in circles
3.		Jah = +su:per *+takes hands down crossing them over the child's feet*
4.	TOM:	*Gazes on C's resting hands*,
5.		(.) *Tom keeps moving arms, touches C' hand*
6.	CLA:	°Mh?° *((intent gaze on child))* (Figure 1b)
7.		(0.3) *Claire smiles*
8.	TOM:	*+Moves hands toward mouth, gazes down to his hands*
9.	CLA:	+Ja: *sup**e:r. *Lifts hands **Takes hands down and crosses them ((similar to lines 1-2, but faster and less wide))
10.	TOM:	**Lays arms down along the body, gaze to C.'s face,
11.		(0.3) *Tom shifts gaze to C's crossed hands and extends his hand to touch them*

**Figure 1:**
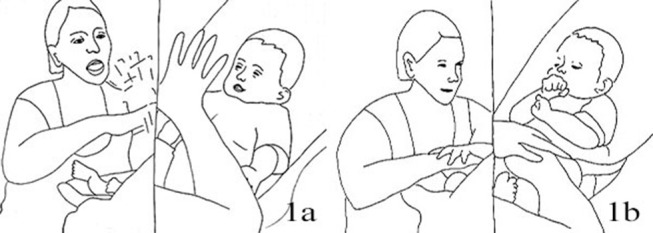
Illustration of Extract 1a. From left to right figures: **(a,b)**.

In lines 1–9 we observe the introduction of the playful movement of lifting up and moving the hands in the air, in front of the infant's face. Two cycles of lifting and taking the hands down occur simultaneously to two repetitions of the “*Ja super”* phrase; the repeated phrases are separated by about 2 s in which the mother gazes at the infant, says “*Mh?*,” then waits some more, her hands resting on the infant's legs. The infant is active at the beginning and keeps his gaze on Claire's hands (lines 1–3); when he becomes reabsorbed in stimuli coming from his own body (line 8), Claire repeats the ‘Ja super' and the hand movement, both toned down with respect to the first time. This re-attracts Tom's gaze and, shortly after Claire's movements have stopped, he looks at her hands and touches them.

**Figure 2:**
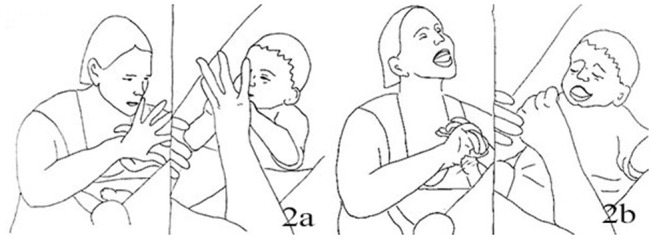
Illustration of Extract 1b. From left to right figures: **(a,b)**.

Claire's turn that includes the verbal “*Ja super,”* coordinated with the hand wave, are prosodically similar with a distinct rise-and-fall shape; however, in their width and intensity they are responsive to the infant's level of engagement (higher first, tossing limbs about and smiling, looking down with the fist in his mouth); both iterations of the phrase are followed by a gap in verbal and body activity from the mother. Figures 1a,b, 2a,b illustrate the excursions in both Claire's turns, from peak (raised hands and sound production) to conclusion. The tempo is one in which each combined verbal and movement phase is approximately as long as the following pauses (lines 6 and 11) in talk and movement, with continuous gaze. Twice in those spaces the infant reaches out for the mother's hands (lines 5 and 11); once his activity displays instead a slight drop in the engagement toward the mother. The mother's actions appear contingent to the infant's, both from a sequential and intonational point of view, either initiating a new turn building up from the infant's touch (line 6) or adjusting to the changed level of the infant's engagement (line 9). In the continuation of the extract Claire takes her hands closer to the infant's face, circling them and waving her fingers for longer bouts of movement than in the previous extracts.

##### Extract 1b

**Table:** 

12.	CLA:	(0.4) *Claire lifts hands, palms wide open*
13.		*°(Warten)°*((Circling r- hand wide open close to the child's face))* *Wait*
14.	TOM:	*Gaze to C's r-hand, Withdraws l-hand, gaze down
15.		(0.5)
16.	CLA:	*Claire waves r- hand's fingers* *^°°^Was ist das^°°^ *What is this* *keeps moving r-hand and fingers
17.	TOM:	*gazes at C's hands
18.	CLA:	*^°°^Guck mal .h::ja^°°^ *Have a look, yes* *Smiles
19.		(1.2)
20.	CLA:	*waves right hand*
21.	TOM:	*Tom mouths hand, gazing to C*.
22.	CLA:	Wenn ich noch das Lied wuesste, *If I just knew the song*
23.		*↑wie das Lied [↑gi::ng = *Howthe song went* *frowns and close hand down in fist (Figure 2a)
24.	TOM:	[Tch((laugh token)) *Keeps smiling wide, shakes head arms and legs*
25.	CLA:	*Jah::a(h)a(h)::: *((with laugh tokens))* (Figure 2b) * shakes head broadly

After Tom touches Claire's hand (end of extract 1a), Claire does a third hand lift, and this time elaborates on the movement, waving one hand and moving the fingers while talking very softly. This lasts about 5 s (lines 12–23), during which the infant moves his arms and mostly gazes at the mother's hands, with what appears as a moderate level of engagement. On the phrase “*Wenn ich noch das Lied wüsste, wie das Lied ging”* (tr. “If I knew the song, how the song went”), pronounced with a higher pitch, Claire stops the hand movement and makes a playful frowning face (Figure 2a); here the infant laughs, and shakes body and head more markedly. To this, Claire responds with a vocal prolonged laugh (line 25) and a broad headshake (Figure 2b). It appears that, when intensifying the infant's stimulation in one modality (the hand wave), the mother plays down the other modalities, speaking softly and maintaining a moderate display of affect with her face. Later, while adding intensity to her voice and facial expression, she retreats the hand, and at this ‘rounding up' of her action the infant laughs and has an excited generalized reaction. Claire's response to this, which mirrors the infant's action (open mouth, body shake), amplifies the infant's agentic move.

In the final extract we follow this dyad to the end of the hand-movement game.

##### Extract 1c

**Table:** 

26.	CLA:	*Wenn ich noch das Lied **wuesste (Figure 3a) If I just knew the song *Reopen hands toward Tom, **waves right hand and circles fingers
27.	TOM:	* keeps smiling broadly with mouth open, gaze to C's face
28.	CLA:	+wie das Lied gi:ng: How the song went *+Withdraws hands*
29.	CLA:	*Ich weiss das **naemlich nicht mehr: *((smiling))* I don't know it anymore *Lifts again right hand in front of T's face
30.	TOM:	**Begins taking hands to mouth
31.	CLA:	*Withdraws hands*
32.	CLA:	* Lifts r-hand again, waves fingers *Hat [die Mama +verge ssen? = Did Mum forget? +*grabs and shakes* *T's wrist*
33.	TOM:	[Ouah:: *+ Turns toward side of the seat, hands in mouth*
35.	CLA	= .Hh[:: *((Smiling))* *lowers hand to take hold of Toms' L hand
	TOM:	[AHh *((Frowning))* *opens l-arm pulling C's hand aside, lifts feet
36.	CLA:	J↑ah↓h:: = *((Goes to neutral face))* *Frees hand and lands them gently on T's feet*
37.		Kannst du schoen wieder = *((bouncing the child's feet))* (Figure 3b) can you do it again?

**Figure 3:**
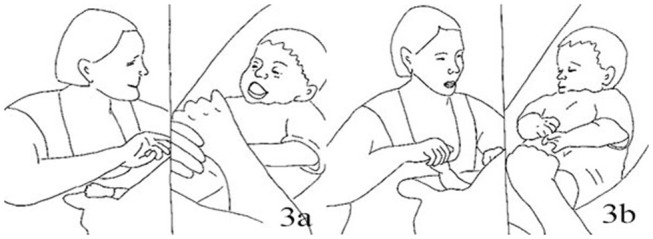
Illustration of Extract 1c. From left to right figures: **(a,b)**.

After the positive reaction of the baby at the hand waving, Claire repeats the movement, briefly, and each time rests her hands down after. Tom keeps smiling broadly at first, with open arms and an intent gaze. On the second lift he starts moving his hand to this mouth, and on the third, which Claire concludes by shaking Tom's wrist, turns both his body and head away, with a frown and a loud vocalization (lines 29–33). Upon Tom's last movements, Claire lowers her hands onto his, but softly, so he has enough strength to move her hand outward. She frees her hands and moves them to the infant's feet. Speaking softly, she pulls his feet up from the toes, and Tom looks at her hands on his feet.

In this last sequence, we have observed the mother performing a hand game she had previously introduced in two short cycles, after the infant's positive affect display, and swiftly reorganizing her movements and body arrangement when the child displayed a change in affect and engagement. When he did so, she distanced the stimuli (the hands) from the baby's body, retreating also with her trunk, and stayed with the baby's feet, still talking to him. The mother's facial expression changed contingently to the changes in the child apparent emotion, softening the smiles into a neutral and at times even “puzzled” expression, which twice in this extract had the effect of moving the child into action.

To summarize, we have seen that the hand-waving as an interactional object was introduced and maintained through several bouts of action, each separated by an interval of relative stillness, in which the mother's facial expression became more neutral and her body stilled; during the resting phases, she kept the gaze on the infant, and was either silent or spoke softly. The mother also acted on the infant's cues, and increased the intensity of her activity as he gave increasing signs of engagement.

#### Episode 2

Extract two also begins less than a minute after the Still-face, and includes a hand waving motion alternated to body contact. At the end of the still phase there had been a momentary distress response, followed by a smile by the infant as the mother talked and held his hands.

##### Extract 2a

MUM: JENNY, INFANT: JACK (3 months) [SG1 M34: 05:02]

**Table:** 

1.	JEN:	*Leans forward *Achtung, *Watch out* * waves RH fingers, thumb still in Jack's LH
2.	JEN:	+Da ist wieder die[Zappelha:nd, *Here comes the wriggling hand* *+ waves fingers*
3.	JAC:	[Ehoh::: (Figure 4a) *looking down at his own hand*
4.	JEN:	(1.0)
5.	JEN:	°Da ist *wieder die <Zappel+ha:nd,°> *Here comes the wriggling hand* *waves RH fingers + *closes palm and starts finger-snapping*
6.	JAC:	*Shifts gaze to waving hand, +let go of Jen's hand (Figure 4b)
7.	JEN:	*Jenny snaps fingers in fast succession, snapping noise hearable* (1.5)
8.	JAC:	+Uh: *+takes LH to mouth*
9.	JEN:	.Hha *((smiling))* (2.0) *Keeps snapping and looking at Jack*
10.	JAC:	*Jack closes eyes, rises fists closed to face, takes other hand to mouth*
11.	JEN:	*Jenny keeps snapping, smiling*
12.	JAC:	+Eh::= *+moves head sideways*
13.	JEN:	=^*^ die Zappel[ha::nd, *((smiling))* *the wriggling hand* *snaps finger
14.	JAC:	[Eh::::: *Brings left hand into mouth*. *Lifts right arm, hand fisted*
15.	JAC:	*Covers face with both hands* *strokes eyes*

**Figure 4:**
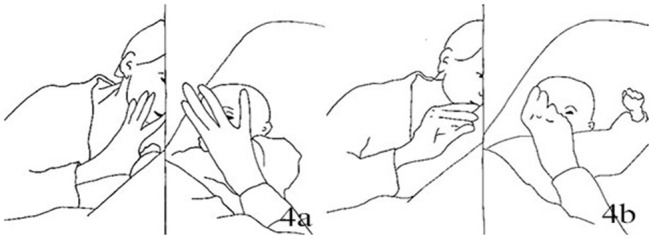
Illustration of Extract 2a. From left to right figures: **(a,b)**.

The hand movement appears for the first time with a finger waving motion when Jack is still holding the mother's thumb, therefore very close to his body and face. The baby seems to be still engaged with his own tactile experience, but at the second repetition of the mother's sentence “*Da ist wieder die Zappelhand”* (tr. “Here comes the wriggling hand”) he looks up to her face and hand. Here Jenny seamlessly goes into the finger-snapping, which has also a sound component. The hand is still in close proximity to the child's face. Jack seems engaged by the new stimulus, as he looks at the hand and moves his arm rhythmically (line 6). About 2 s into the finger snapping the infant vocalizes and moves his hand toward the mouth (line 8), a well-known self- soothing behavior in infancy. Jenny reacts to the vocalization as if it was a positive reaction to the game, saying a sort of aspirated “*Ja”* (Yes) and smiling more deeply, while continuing finger-snapping. The infant takes his other hand to mouth then both hands to his face, vocalizes in a more distressed tone and moves his head away. Jenny continues finger snapping and smiling, and repeats ”*Die Zappelhand,“* until Jack utters a third vocalization, now longer and sounding more distressed, with more hand movements covering the face. Here Jenny stops her movement to inquire about his seemingly changed mood (“*Was”*–“What,” line 16). We discuss briefly this segment before looking at the resolution of this episode.

Jenny's hand movements have been continuous, with no rest phases in between; her facial expression was steady, smiling throughout although with some variation in intensity. Her trunk was leaning over toward the baby for the whole time, and the movements of the hands performed in the proximity of the infant. None of her gestures are *per se* bound to create discomfort: at different times in the interaction the infant reacted to them with either interest or with withdrawal. However, there is an absence of any kind of ‘pulse' in the activity, of the kind we observed in extract 1a,b, and c. The steadfast progression and display of the same affect configuration appear distressing for the infant already a few seconds into the episode. He becomes louder, the hands in the mouth or covering the eyes, with long-held frowns. The pattern that seems to be emerging here is that of a stimulation that is held until the infant ‘breaks free' of it: when eventually there are clear signs of discomfort and withdrawal, the mother's actions become more clearly responsive and contingent.

##### Extract 2b

**Table:** 

16.	JEN:	Wa::s. *((baby-talk voice))* *What* *((stops snapping and takes hold of Jack's hand))*
17.	JAC:	*Crosses arms over face, palms out* [Uh:: *((frowning))*
18.	JEN:	[(Ha*st du kei ne lust mehr) *((soft))* *You don't fancy it anymore* *Crawls fingers across Jacks' chest
19.	JAC:	*+Looks at Mum over raised fisted hands* +Eh:
20.	JEN:	Was du [ka:nnst*, ((moving fingers over J's chest))* *What you can*
21.	JAC:	[He::::*:: *Looks away
22.	JEN:	No:::
23.	JAC:	*Takes gaze back on Jen*
24.	JEN:	Oh: bist du +m(h)u:de *Oh are you tired* *+Takes both Jack's hands in hers moves them outward*
25.	JAC:	*Uhgh *Pulls left arm away, hand breaks free from Mum's hold
26.	JEN:	+Bisst du vielleicht [mude (Figure 5a) *Are you maybe tired* *+Pokes J.'forehead with index of right hand, left hand still holds J's left*
27.	JAC:	[He:: (Figure 5b) *((Covers face with both hands, taking right hand out of Jen's hand))*

**Figure 5:**
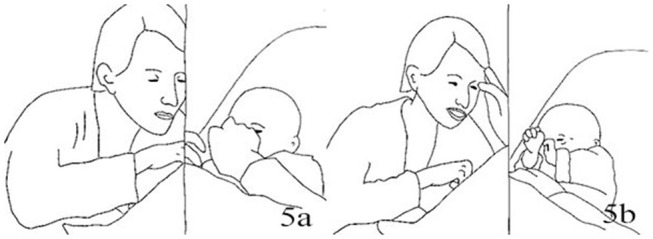
Illustration of Extract 2b. From left to right figures: **(a,b)**.

Jenny's words (“*Hast du keine Lust mehr,” tr*. “You don't fancy it any more” and “*Oh bist du müde,” tr*. “Oh are you tired”) reveal that she has noticed the mood change and interprets it as related to the hand game. She does not, however, completely pause the infant's stimulation: she stops the hand movement but takes hold of the infant's hands. Then, still holding Jack's hand, she moves her fingers across his body (lines 18 and 20). The infant gazes back to the mother and stops vocalizing, but does not lower his fists and looks at his mum from above his own hands, still covering most of his face. There is a sense of the infant not ‘lowering his guard' here, not completely letting go of the body tension and cover. After this, Jenny holds Jack's hands and opens his arms outward, to which Jack abruptly throws his left arm up, letting go of the mother's grip. The mother acknowledges these actions as indicating trouble (line 26) and keeps soothing him by keeping his hand in hers and poking gently the infant's forehead with the other, leaning closer. Again, the infant reacts to the mother's voice positively, looking at her and briefly smiling, keeping his body leaning on one side, his face partially covered, and vocalizes with a distressed tone.

Worth noticing, Jenny has kept smiling throughout, her expression not being modulated by the infant's changes in affect. There is a constant flow of physical touch in this interaction, so that the pauses between verbal utterances do not create a segmentation in the mother's activity. It is worth emphasizing that the hand movement and touching of the infant's body are the primary modality of communication in these examples (Bremner and Spence, 2017), and therefore the one in which the different rhythms manifest with more salience for the child.

#### Comparing Episode 1 and 2

Comparing analyses of episodes 1 and 2, it becomes visible that properties of the mother's action that seem more significant in terms of positive vs. negative engagement relate to duration and rhythmical quality of the actions, rather than to the movements *per se*. In episode 1 the rhythm of mother's actions toward the infant—hand waving and touching—was made of cycles, the end of each marked by a change in affect and body distancing. Not only metaphorically, but also literally the infant was given space during the intervals; there, he would rearrange his limbs, inspect the mother's expression and gaze, and either perform bids for re-engagement or self-centered actions, giving the mother the opportunity to adjust her successive actions accordingly.

In the second episode, the infant interactional and physical space was more constrained. Separate bouts of talk from the mother happened on the background of continuous, very prominent hand movements; the baby's own movements and vocalization overlapped with hers, ending up being somewhat absorbed within them, until a more patent withdrawal was performed. Even after the infant clear distressed reaction, the mother maintained physical proximity and active touching, mirrored by the physical protective posture of the infant. Interestingly, the infant's agency was supported more when he expressed negative affect. This sustained interactional rhythm, in other words, while stimulus-rich and apt to involve the infant in dyadic communication, included fewer opportunities for him to notice the effects of his activity and re-experience some of its qualities as reflected in the mother's mirroring of them (as happened in episode 1).

Finally, a feature of the first episode we have not touched on above is the introductory phase in which the hand game had been performed with less intensity and proximity (the hand circling movement was performed in shorter repetitions and distant from the child's body). Upon the infant's attention and engagement, the mother developed the movement, sustaining it after for a longer time and going closer to the infant's body and face. The introductory phase contributes to creating a smooth interaction in which an activity that might have been considered intrusive in isolation, is familiarized to the child slowly but progressively. This phase was missing in the second episode in which a rather intense hand game from the point of view of duration and proximity had started abruptly in its full form. The importance of preliminary phases in infant-directed activities will be expanded in the following paragraph.

### Preliminaries

The episodes presented below show two different dyads engaging in a similar series of playful lifting/pulling up sequences. In both episodes, the mother's action (holding of infant's wrist and pulling up movement) had been coded as intrusive according to the ICEP-R criteria. However, while the structure of episode 3 includes a preliminary sequence to the pull up action, in episode 4 the structure is flat, i.e., the repetitions of the pull-up movement are similar to each other, without a discernible sequential development.

#### Episode 3

Episode 3 begins with the infant seated in a babyseat and the mother on a chair in front of him, both looking at each other.

##### Extract 3a

MUM: AMY, INFANT: MIKE [SG1 M106 00. 04.54–00.05.02]

**Table:** 

1.	MIK:	*Oh:huu+:: = *((smiles))*	
2.	AMY:	*Smiles, holding M. hands loosely	
3.	MIK:	*+((Chin up, gazes away))*	
4.		= Ga:ga:: =	
5.	AMY:	= Eh:blublu**blu::= *((Smiles and nods))*	
6.	MIK:	**Gazes at M.	
7.		(0.2)	
8.	MIK:	*Arches back, chin up, legs tucked up* (Figure 6a)	
9.		= [*Mh:h:o:ugha:: *turns head right, gazes away	
10.	AMY:	Oh: +Willst du aufste*::hen? *Oh would you like to come up?* + *draws M's hands toward her*	Preliminary opening
11.	MIK:	*Gazes back to A.	
12.		*Makes brief lifting up attempts and gazes away* (0.9)	
13.	AMY:	*Willst du aufste::*hen?= *Would you like to come up?*	
14.		*Slowly pulls the infant's hands up	
15.	MIK:	= Ouhaa:: =^**^((smiling))	
16.		**((Tucking legs up, arching back and moving chin up))	
17.	AMY:	= Komm wir ueben mal= *Let's have a go*	
18.	MIK:	* Lifts all the way up till sitting straight, head close to A.	
19.	AMY:	= ↑Ahh::genau:u*hm:mm::hmm::((laughs)) *Ahh that's it((still holding M's hands))*	
20.	MIK:	*++ Reaches an upright position with forehead close to A.'s lips*	
21.	AMY:	*++Kisses M. on the forehead* (Figure 6b) (0.5)	
22.	MIK:	*Starts moving back toward babyseat*	
23.	AMY:	Hm:m::supe[:r::.↓	
24.	MIK:	[Hm:ga:aa ↑	

**Figure 6:**
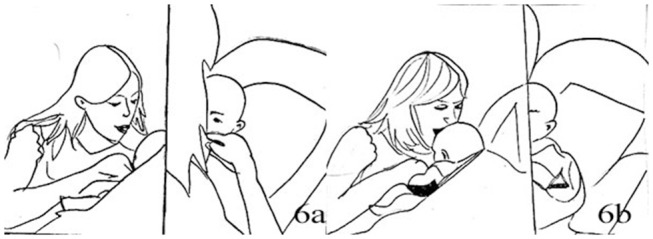
Illustration of Extract 3a. From left to right figures: **(a,b)**.

The episode starts with Mike, the infant, and Amy, the mother, smiling while looking at each other. Mike then makes a vocalization (line 1) and gazes away. Amy imitates Mike producing a similar sound, still smiling, and Mike briefly gazes back at her. He then makes postural adjustments (arches back, chin up, legs tucked up) which are treated by Amy as a preparatory initiative to lifting himself up. She aligns with Mike's attempt by verbally formulating it (“*Oh, willst du aufstehen?*,” tr. “*Oh, would you like to come up*?,” line 10). After a brief pause, when the infant orients back toward the mother by looking at her and moving his head, Amy repeats her question with a more marked ascendant intonation accompanied by head nodding ((“*Oh, willst du aufstehen?*,” tr. “*Oh, would you like to come up*?,” line 13). To this, Mike makes a soft vocalization and smiles. Amy then reinforces her previous comment by making an explicit invitation for jointly acting (“*Komm wir ueben mal*,” tr. “*Let's have a go*,” line 17). Before the end of Amy's turn Mike lifts himself up on its own strength, adopting the same pattern of movements used previously. Amy, holding the infant' hands, makes a coordinated action which complements the infant's movement by sustaining his lifting action toward her. She accompanies her movements with a bright smile and a positive assessment of the infant's initiative (“*Ah, genau,”* tr. “Ah, that's it,” line 19). Once Mike is completely upright and close to Amy's face, she makes an additional non-verbal preliminary move toward the accomplishment of the first part of the action (that is, the end of the lifting movement), by leaning forward and kissing the infant on his forehead (line 21). After that, the infant slowly releases his body moving back toward the babyseat. Amy holds the infant's hand throughout this second part of the interactional sequence. Once Mike is almost lain down, the mother makes a comment to mark the closing of the lifting/pulling-up sequence in the form of a positive verbal assessment (“Super,” line 23) to which the infant smiles broadly. This comment may serve as preliminary announcement of the closing of activity, which is accomplished by the infant returned in his original position and the mother oriented toward him, smiling and holding his face.

Between this initial episode and the one presented below (extract 3b) four more pulling/lifting activities occur, each initiated by a movement of the infant and followed by a metapragmatic comment of the mother inviting the infant to repeat the activity once more (“*Nochmal?* “*tr*. “*Again*“). For two times the mother makes a preliminary announcement of the forthcoming action verbalizing her counting (“*Eins, Zwei, Drei”* tr. “One, two, three”) before starting the pulling/lifting activity. The closing of each sequence of actions is accomplished by a positive verbal assessment like “*Sehr gut*” (tr. “Very good”) or “Super.”

In their last lifting/pulling sequence (Extract 3b) the mother and the infant seem to negotiate the end of the interaction by marking its completion (the mother), disengaging from the interactional space (the infant's turning the head away) and both displaying a neutral face, in contrast to the overall positive affect display that had characterized their facial expressions up to this point.

##### Extract 3b

**Table:** 

25.	MIK:	Ohga:↓[ra::: = *((gazing at A., neutral face))
26.	AMY:	*Smiles still holding M.'s hands
27.		(1.4)
28.		[↑Ohr::aga:↓the:**e: = *((nodding, neutral face))*
29.	MIK:	**Frowning expression
30.	AMY:	= Ah*h↓:: *((Neutral face, nodding))*
31.	MIK:	*Mouth open widely, neutral face
32.		(2.0)
33.	AMY:	+Jah**:: ((soft))
34.		**Protrudes lips out as to kiss M. (Figure 7)
35.	MIK:	*+ Starts lifting up toward A*.
36.		*++ Upright but still moving toward A*.
37.	AMY:	*++Kisses M. on the head* (0.5)
38.	MIK:	*Slowly moves back toward the babyseat* (1.0)
39.		* Lain on the babyseat *((head turned on the right side, gaze to A. sucking her hand))*
40.	AMY:	*Gazes at M. with neutral expression (Figure 8)
41.	MIK:	*Turns head away*

**Figure 7:**
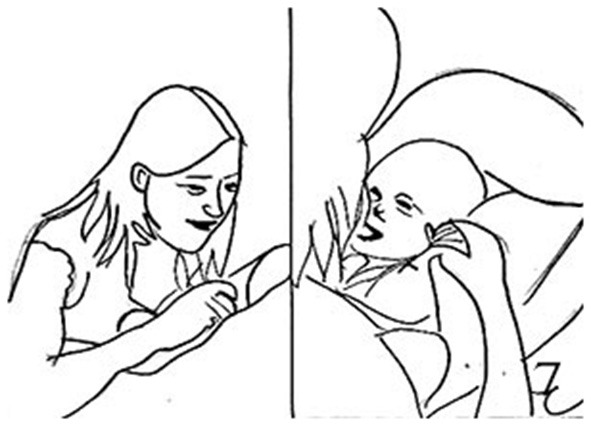
Illustration of Extract 3b.

**Figure 8:**
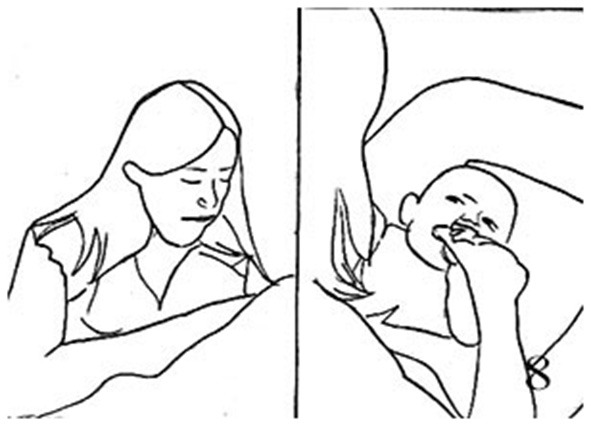
Illustration of Extract 3b.

The sequence begins with a downturned vocalization of the infant displaying a neutral face (line 25). The mother then aligns with Mike's affective display at two levels: she acknowledges the infant's vocal production by repeating it (with the same intonation) and yet, at the same time, she downgrades her affective display from positive to neutral (line 28). After that, she multimodally aligns with the infant's affective display by making a vocalization (“ah”) with a descendent intonation (line 30). After a brief pause, the infant then starts lifting himself up. The mother acknowledges this attempt as an invitation to do an additional cycle of pulling/lifting activity and responds to it with a positive assessment (“*Ja*,” tr. “Yes”), yet in a whispering monotone way, mirroring the infant's affective tone. While the infant is still moving up and has not yet completed his lifting movement, the mother protrudes her lips in a kissing-shape and then leans forward to kiss him on the forehead, catching on the infant's forehead when he is not completely upright, but still moving. She thus anticipates the closing of the action, pre-announcing the end of the entire cycle and overall activity of the pulling/lifting kind. After his mother's kiss, Mike moves back onto the babyseat, suckling his mother's thumb with a neutral expression, and eventually turning his head and gaze away. Amy keeps looking at him, yet displaying a neutral expression, with downturned lips. The activity is finished.

In the extracts just described the infant's participation is facilitated by an overall clear sequential structuring of each pulling/lifting sequence, guided by the mother who builds up each successive move on to the infant's previous one. Particularly relevant in this episode is the use of multimodal preliminary moves by the mother to prepare the ground for the next relevant action, being it the opening or closing of the activity. Preliminaries, also defined as *pre-sequences* (Schegloff, 1980), are a generic term for a class of conversational moves projecting what comes next. They are specific to the type of conversational content following them (e.g., pre-invitation, or story preface) and include a form of acknowledgment from the recipient. In this episode, Mike's vocalization and lifting up movements (line 18) may be seen as an acknowledgment of the mother's preliminary invitation to perform a joint lifting activity. The closing of the sequential pulling up activity is also systematically and multimodally anticipated by the mother through a kiss on the infant's forehead (as in lines 21 and 37), followed by a positive verbal assessment to mark the (successful) completion of the activity.

#### Episode 4

The following episode also revolves around a pulling/lifting sequence with infant and mother as participants, yet important differences with the previous episode emerged during the analysis. Due to limited access to the original video, this episode is presented in a different “format” compared to the previous ones. The analytical approach (CA oriented) remains nevertheless the same.

##### Extract 4

[Mum: Sara, Infant: Jim [SG1 M3 00.10.50–00.13.6]

**Table:** 

	**JIM**	**SARA**
1.	Right arm raised toward the mother, body slightly leaning forward (Figure 9a)	Looking at J., smiling with close lips
2.	Lowers arm down, looking at S., body and head partially oriented on the opposite side	“Was willst du machen?What do you want to do?
3.	(3.0)	Smiling and gazing at J. holding J. by the arms,
4.		Starts opening J.'s arms broadly
5.		Starts pulling J. toward her by holding his hands
6.	Arms broadly open toward S., starts pulling up legs	Keeps pulling up J. with slow movements
7.	Arms flat and outstretched	
8.	Body pulled up forward toward S. with no tension, back arched as the buttock is still on the babyseat while the arms are outstretched (Figure 9b)	
9.	Lifting toward S. with close eyes	Keeps looking and smiling at J.
10.	Head facing down, trunk bent and fastened forward by the arms	
11.	Stops moving	Pauses pulling up movements.
12.	Still, head facing down at S.'s breast level; gaze down.	
13.	Moving backward toward the babyseat, arms held by the mother (Figure 10)	Sustaining J's backward movements.
14.	Head turned on the right side, laying on the babyseat, eyes closed	Keeps holding Jim by the hands, gazing and smiling at him.
15.	Leaning back on the babyseat, face oriented toward the mother (1.6)	
16.		Gazes at I, neutral face

**Figure 9:**
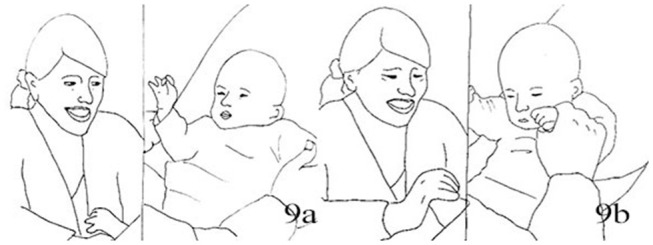
Illustration of Extract 4. From left to right figures: **(a,b)**.

**Figure 10:**
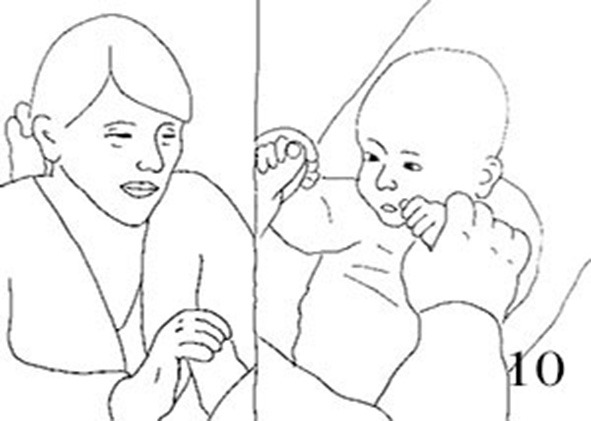
Illustration of Extract 4.

At the beginning of this episode, Jim and Sara are oriented toward each other. Sara displays a close-lips smile while Jim has a neutral face. Jim then raises the right arm toward the mother, while the whole body is slightly leaning forward. He keeps a steady gaze on to the mother, holding this position for about 3.5 s. The mother acknowledges the infant's movement of the arm making a verbal comment with a low, un-modulated tone (“*Was willst du machen?*,” Tr. “What do you want to do?). This comment presents a rather different pragmatic quality compared to the one made by the mother in episode 3. Here, the mother is not asking the infant specifically what he's doing. She does not verbally reformulate the infant's movement but rather makes a comment, which seems to mark the un-specificity of the infant's move, implying that this was not understood by the mother. A pause of about 3 s follows (line 3), during which the mother is looking at the infant, smiling, while the infant's body and head is partially oriented on the opposite side, yet still looking at the mother. Despite acknowledging the infant's initiative, the mother does not build upon it, leaving an empty interactional space though maintaining her engagement with the infant by keeping smiling at him. After this pause, she closes her hands on the infant's wrists, opens up his arms and moves them up. Then, she suddenly begins to pull the infant toward her (line 5) by holding him on the wrist. Since there is no clear announcement of the mother's intention to pull the infant's up, nor is there any behavioral or verbal sign marking the beginning of the action (with the exception of the mother's holding and opening the infant's arms), Jim appears as not fully prepared to join in Amy's move. Indeed, his body is passively pulled forward by the mother rather than actively lifting up on its own strength. Such passivity is visible in his arms, outstretched but not tensed; similarly, Jim's trunk appears to bear no forward tension. Jim's head is bent forward, embedded into his outstretched arms, hindering the possibility of looking at the mother or being looked at on the face (line 10). When Jim is completely lifted up, the physical space between him and the mother is still considerably wide, as if the lifting was not fully accomplished. Then she slowly moves Jim back toward the babyseat. During the descending movements his head is leaning sideways and floppy, while Jim is not looking at the mother but at the side of the room.

Two more similar sequences follow lasting respectively, 2.5 and 3s, each coming quickly after the previous one with almost no pause in between. The movement coordination of pulling (by the mother) and lifting (by the infant) sequences seems to improve over time. Jim's arms are bent and not outstretched, the head is sustained upright and the back position is not arched anymore. Similarly, the mother's body is also less tensed, as if the efforts in pulling the infant's up were slowly decreasing. While after each lifting/pulling sequence the dyad is more coordinated, the affective quality displayed by the dyad in this interaction remains dis-aligned—the mother displaying a still, un-modulated smile and the infant showing a still, neutral facial expression. There seems to be no progress or modulation in the affective engagement of Jim and Sara.

#### Comparing Episodes 3 and 4

Although presenting a similar pattern of actions, our microanalyses have revealed important differences in the quality of engagement and modalities of infant's participation between the episodes just described. In both episodes the mothers make the infant's initiative explicit by verbalizing it, and then acting upon it to build up an entertaining and co-constructed interactive exchange (Berducci, 2010). However, only in episode 3 these initiatives are also quickly reused by the mother (lines 9 and 13) to formulate an invitation for engaging in a shared activity (line 18). The presence of preliminary moves by the mother contributes to a broader clear structuring of the activity as a defined event, presenting clear marking of boundaries such as openings and closures, introduced by preliminary moves which make the activity more visible and predictable for the infant. The use of a preliminary sequence, accomplished by a combination of multimodal actions, seems to be of critical importance for establishing a shared orientation with the infant before the activity begins.

On the contrary, but in accordance with the key interactional aspects emerged in episode 3, Jim's lack of preparation evident in episode 4 seems partially related to a delay in the mother's contingent alignment with the infant's activity, at least at the beginning of the sequence (a similar phenomena was described by Fantasia et al., 2016). The poor quality of Jim's engagement might be accounted for by a lack of sequential structuring of the sequence, of the kind described above. The affective dis-alignment emerged in episode 4 might be related to a maternal difficulty in contingently responding to the infant's feedback. The infant's initial movement of the arm is not promptly acknowledged by the mother, becoming a missed opportunity to use the infant's initiative as a preliminary marker for the beginning of the lifting/pulling sequence.

The presence of pauses within and in between each lifting/pulling sequence is an additional aspect of the interactional organization differentiating the two dyads observed. Pauses, silent gaps between interactants, may have different functions depending on their duration and place in the interaction. They create spaces of no-action where participants can change or repair the ongoing activity, alternate the speaker's turn or signal an intention to end the interaction (Jefferson, 1988). At the end of extract 3, for instance, a very brief pause marks the closure of the interaction, as both the infant and the mother do not upgrade or relaunch the activity but rather let it gradually die out. In episode 4, on the contrary, the fast succession of lifting, one straight after the other, does not leave such spaces, offering little opportunities for the infant or the mother to smoothly introduce variations to the ongoing activity (in this sense, extract 3 echoes extract 1 in the importance of rhythm in the delivery of engagement bids from the mother).

Finally, the affective quality emerging from the analyses differs noticeably between the two episodes. In episode 3, mother and infant display a variety and modulation of affects, both positive and negative, shared as participants align with each other's affective state and leading to an increasingly playful quality of the lifting/pulling sequences, or a gradual shared disengagement, as showed in extract 3b. In episode 4, despite a visible improvement in the coordination of pulling and lifting sequences over time, there seems to be almost no progression in the quality of affects, neither at the level of individual's affective display nor at the interactional level. In other words, both the mother and the infant present a frozen affective quality during the course of the interaction expressed by the infant's neutral face and the mother un-modulated smiling.

## Discussion

The aim of the work presented in this paper was to explore the interactional dynamics underlying maternal behaviors previously identified as intrusive by mainstream literature on postpartum depression. To do so, a conversation analytical approach was adopted to analyse a small sample of clinical and non-clinical mother-infant dyads, observed during a Still-face experimental procedure. Two aspects concerning the sequential structuring emerged in the analyse accounting for the affective differences of the dyadic engagement: *interactional rhythm* and *preliminaries*.

In episodes 1 and 2 *interactional rhythm* emerged as a discriminatory dimension in the occurrence of resting phases between consecutive action bouts, and relative modulations on the affective configuration both in conjunction with the caregiver's own action and as a response to the child's variations. Comparing episodes 1 and 2 has shown that properties of the mother's action that seem more significant in terms of positive vs. negative engagement actually relate to duration and rhythmical quality of the actions, rather than the single movements *per se*. A longer activity time was functional for slowly but systematically integrating the infant's moves within the sequences of activity. Similarly, the presence of an introductory phase contributed to smoothen the interaction, insofar as an activity that might have been considered intrusive in isolation (waving hands closely to the infant's face) is familiarized to the infant slowly but progressively. This phase was missing in the second episode, where the modulation of the mother's own ‘presence' over time as a combination of physical proximity, touch movement and vocal stimulation was not aligned with the infant's affective feedback. Interactional rhythms characterized by continuous stimulation and absence of sequential boundaries can lead to an imbalance in the interactional participation, as highlighted in episode 2.

In episode 3 the presence of preliminary moves accomplished by a combination of multimodal actions appeared as a central element giving visibility to the sequential organization of the activity. In adult conversation, preliminaries are utterances that help listeners understand the trajectory of the talk and be responsive in pertinent places (Schegloff, 1980, 1988). In our analyses, they seem to fulfill two main functions: a) creating a shared focus of attention, orienting the infant toward the mother before any activity begins and 2) making the next interactional turn more predictable for the infant, helping him to “anticipate a “now” moment and to coordinate actions with another” (Goodwin, 2017:84). Preliminaries, along with the presence of pauses within and between sequences of activity, are part of the mother's practice of encapsulating each lifting/pulling sequence into a defined event, with a clear opening and closure, aligning not only with the infant's action but also with his affective tone. In this way, an overarching frame for the single but jointly constructed activities is provided, shaped in a narrative-like excursion developed over time. In episode 4 the engaging attempts by the mother are also expressed in the building up of pulling/lifting sequences taking into account the infant's initial pulling up attempt. The fast pace with which each of these sequences follow one after the other, however, underlines how the poor sequential structuring seems predictive of temporal and affective dis-alignments, as the possibilities for the mutual coordination of actions and affects are limited.

The findings emerged from our observations have two main implications. First, they call for a serious reflection on the theoretical assumptions and methodological practices endorsed by mother-infant research, especially that involving clinical participants. Our analyses uphold the main criticism advanced in the introduction section toward accounts of intrusiveness considering individual actions in isolation, or even in a single action-response sequence, as indicative of a felicitous or less felicitous interaction. Actions such as holding the infant's hand, pulling, physically invading the infant's space are necessary *maneuvers* to commence a new action, or responses to the infant's initiative, as emerged in episodes 3 and 4. They are part of the way everyday situations are accomplished and regulated by adults, who perform actions with and on infants, without whom infants would not survive. Although theoretically grounded or inspired by the Mutual Regulation Model, mutuality and affective coordination seem to have moved to the background in previous studies adopting time-series analysis of single behaviors to assess dyadic engagement. In these studies, the focus of investigation has therefore shifted from how mother and infant mutually coordinate to what the mother *does* with or on the infant, assuming that all maternal behaviors are initiatives (such as cutting across or interrupting the infant's action) instead of responses or attempts to align with the infant's communicative signs. Within this view, the infant then becomes a recipient for someone else's action instead of being a participant in a shared activity. On the contrary, a CA approach considering sequences of activities as analytical units supported an evaluation of the interdependence level of each partner's respective acts, supporting a clearer picture of the interactional sequences facilitating or restricting the infant's participation.

Secondly, our analyses suggest that what seems to be predictive and discriminative of positive and negative interactional outcomes of similar actions is the normative organization pertaining to the order and structure of the interactional sequence. Analyzing longer sequences including iterations of an act or pauses enabled the consideration of timing and variations in intensity across repeated actions, as well as the identification of interactional rhythm and sequential organization (including preliminaries) as important aspects of this normative organization. Although they have been identified here as analytically separate, these aspects are nevertheless part of the same dialogic and interactional dimension whose structure became more visible thanks to the robust and reliable conversation analytic framework. Although limited to only four examples, our findings seem to complement recent empirical evidence that even very young infants are able to understand and anticipate well-known self-directed actions (Reddy et al., 2013) particularly when these actions present invariants in timing and sequential structuring (Fantasia et al., 2016). The clear structuring of episode 1 and 3, sustained by the presence of preliminaries and transition spaces may be seen as a foundational framework within which infants make experience of this emerging capacity, progressively gaining more resources and chances to be a partner in different types of interactional formats.

A brief methodological consideration on the clinical as well as analytical implications of using the Still-face paradigm for testing mother-infant spontaneous interactions can be advanced. During the Still-face procedure a strict spatial configuration is imposed on to participants: mother and infant are positioned face to face, with the infant still (literally) fastened in the babyseat and the mother seating in front of her. No objects or toys are allowed. As a result, while the mother can make use of a variety of interactional resources (with some limitations, for instance she cannot stand up or get the infant off the babyseat), the infant has very little room for moving, or changing activity. In other words the mother has possibilities for initiating or maintaining the activity that are instead limited in the case of the infant, leading to e.g., unbalanced proportions of actions performed within the dyad as increased number and variety of actions by the mother. Differences between depressed and non-depressed mothers have been observed mainly in the reunion phase of the still-face (Weinberg et al., 2006); the extremely challenging nature of this phase should be thus taken into consideration when interpreting the different outcomes of the episodes analyzed in this paper.

A final remark in the present discussion is needed to stress that this study did not aim to compare the quality of interactions in two populations (i.e., depressed vs. non-depressed mothers), but rather to unpack the interactional dimensions at play in episodes identified as intrusive under mainstream descriptions. Whether these interactional patterns characterize mothers with psychological difficulties more than non-clinical mothers, and whether they extend over the first months of an infant life is for further research to establish. Although it is not possible to ignore that these aspects were played differently by the mothers diagnosed with postpartum depression, this difference may be due to a variety of factors, including the pressure of the experimental condition (higher for clinical mothers), which may have induced PPD mothers to overstimulate the children or keep an “upbeat” attitude throughout.

We are aware that the analysis of episode 4 present a far less accurate and fine-grained level of details, affecting the overall analytical power and interpretation not only of that specific episode but also of the conclusion we have attempted to draw. The strength of our claims in this work has been calibrated accordingly.

To conclude, the findings provided by the present study may be considered one step on the way to the development of new conceptualizations, ethnomethodologically oriented, that would inform the theory and method of future research in clinical and non-clinical populations. Although the boundaries between stimulating and restricting are not easy to draw, our analyses have shown a central weakness in the very idea of intrusiveness that is not resolutive but opens new questions, such as: how do we distinguish between behaviors which are positively stimulating the infant and others that are undermining their autonomy? And what can be the meaning of ‘autonomy' in the context of the infant's action? We feel that future research focusing on the development of infant's capacities to participate in orderly, sequential interactions should take these questions into account.

## Data Availability

All datasets generated for this study are included in the manuscript and/or the supplementary files.

## Ethics Statement

This study is based on the analysis of already collected data from a previous study. Ethical approval from the University Medical Faculty, Heidelberg was granted for the original study. In this paper we maintain the anonymity and confidentiality of the data.

## Author Contributions

VF, LG, and AF analyzed the data and wrote the paper. CR provided the data and revised the paper.

### Conflict of Interest Statement

The authors declare that the research was conducted in the absence of any commercial or financial relationships that could be construed as a potential conflict of interest. The reviewer MF declared a shared affiliation, with no collaboration, with one of the authors VF, to the handling editor at time of review.

## Appendix A

### Transcription Symbols

Adapted from Jefferson (2004)

**Table:**

:	Colon(s): Extended or stretched sound.
__	Underlining: Vocalic emphasis.
(.)	Micropause: Brief pause of less than (0.2).
(1.2)	Timed Pause: Intervals occurring within and between same or different speaker's utterances in tenths of seconds.
(())	Double Parentheses: Contextual information.
(don't/won't)	Single Parentheses: Transcriptionist doubt (best guess) or (guess/other guess).
.	Period: Falling vocal pitch.
?	Question Marks: Rising vocal pitch.
!	Exclamation Points: Animated speech tone.
WORD	Caps: Extreme loudness compared to surrounding talk.
°°	Degree Signs: A passage of talk noticeably softer than surrounding talk.
[	Brackets: Marks the beginning point at which current talk is overlapped by other talk.
*	or + Mark simultaneity of actions in two consecutive lines
↓↑	Arrows: Pitch resets; marked rising and falling shifts in intonation.
=	Equal Signs: Latching of contiguous utterances, with no interval or overlap.
><	Less Than/Greater Than Signs: Portions of an utterance delivered at a pace noticeably quicker (> <) or slower (<>) than surrounding talk.
∙	Hyphens: Halting, abrupt cut off of sound or word.
.hhh:	Audible inbreaths
h h:	Audible outbreaths from such events as laughter, or sigh
wo(h)rd(h)	outhbreaths within words
